# Lumbar disk herniation surgeries: the impact of preoperative holding area duration on anxiety and postoperative pain

**DOI:** 10.55730/1300-0144.6079

**Published:** 2025-08-28

**Authors:** Zuhal KOÇ APAYDIN, Hakan KINA

**Affiliations:** 1 Department of Psychiatry, Karabük University, Karabük, Turkiye; 2 Department of Neurosurgery, İstinye University, İstanbul, Turkiye

**Keywords:** Preoperative anxiety, postoperative pain management, preoperative holding area waiting time

## Abstract

**Background/aim:**

The purpose of this study is to investigate how anxiety levels are affected by preoperative holding area waiting times and how these fears affect pain following surgery. Patients waiting for surgery frequently suffer from preoperative anxiety, which can have an impact on how well they manage their postoperative pain.

**Materials and methods:**

Forty-eight individuals with lumbar disc herniation scheduled for surgery participated in the study. Patients’ anxiety and pain levels were measured both during and after the preoperative holding area waiting period using the Amsterdam Preoperative Anxiety and Information Scale (APAIS), State-Trait Anxiety Inventory (STAI), and Visual Analog Scale (VAS). Additional information was also documented, including waiting times and tramadol usage.

**Results:**

The APAIS (postwait) score and preoperative holding area waiting time had a significant correlation (r = 0.669, p < 0.001). Initially, a positive correlation was found between waiting time and postoperative VAS scores (r = 0.309, p = 0.033), as well as between the tramadol dosage used for postoperative pain management and the STAI-S (postwait) and STAI-T scores (STAI-S (postwait): r = 0.286, p = 0.046; STAI-T: r = 0.306, p = 0.035). However, after applying the Bonferroni correction for multiple comparisons, these associations no longer remained statistically significant.

**Conclusion:**

Patients’ anxiety levels rise with longer preoperative holding area waiting times, and this has an impact on their postoperative discomfort. Increased preoperative anxiety is associated with greater postoperative analgesic requirements, whereas reducing preoperative anxiety may enhance patient satisfaction and surgical outcomes. Postoperative pain and recovery may be enhanced by cutting down on waiting periods, educating patients about the surgical procedure, and putting anxiety management strategies into practice.

## 1. Introduction

One of the most frequent problems that patients who are having surgery deal with is preoperative anxiety. It is frequently described as a state of tension and discomfort arising from patients’ uncertainties or fears regarding the procedure [1]. Among the most common causes of preoperative anxiety are waiting for the surgery, concerns about the outcomes of the procedure, anticipation of postoperative pain, fear of losing independence, and fear of death [2]. Various factors associated with preoperative anxiety include psychosocial variables, previous surgical experiences, having information about the surgical process, and anesthesia-related concerns [2–5]. Strong evidence indicates that preoperative anxiety can affect responses to anesthesia and analgesia [6] and is significantly linked to postoperative pain [7]. Additionally, anxiety not only elevates postoperative pain levels but can also lead to depression, nausea, and fatigue; impede wound healing; and ultimately delay hospital discharge [8]. Research by Tadesse et al. [9] and Ali et al. [10] has shown that patients experiencing high preoperative anxiety report greater postoperative pain levels in the first 12 h. Similarly, Wu et al. [11] and Deng et al. [12] demonstrated a positive correlation between perioperative anxiety and pain levels in patients undergoing breast surgery.

In recent years, there has been growing interest in interventions aimed at reducing preoperative anxiety, as this condition has been associated with increased postoperative analgesic requirements, prolonged hospital stays, and delayed recovery processes [13,14]. These interventions can be classified as pharmacological, typically involving sedatives and anxiolytics, and nonpharmacological. However, concerns regarding potential complications such as respiratory issues, interactions with anesthetic drugs, and delayed postoperative recovery associated with pharmacological interventions [15,16] have led clinicians and researchers to explore nonpharmacological alternatives.

Nonpharmacological interventions include preoperative counseling and education, religious or spiritual activities, aromatherapy, music therapy, and acupuncture [17–21]. Among these, studies have found that effective communication between the surgeon and the patient is the most impactful method in reducing preoperative anxiety [22]. Other nonpharmacological techniques shown to alleviate anxiety include religious/spiritual practices before surgery [23], visual and auditory educational videos about the surgical procedure [24], massage [25], and acupuncture [26]. However, findings regarding the effectiveness of aromatherapy in reducing preoperative anxiety remain inconsistent and conflicting [27–29]. Such approaches are particularly valuable due to their low cost, ease of implementation, and absence of drug-related side effects. However, these interventions may not be feasible in all healthcare settings, as they require specific planning and resources.

These studies, however, evaluated preoperative anxiety either on the day of the operation or at least 24 hours prior to surgery. Acute preoperative anxiety in the preoperative holding area has been investigated in relatively few studies [30,31]. Most of these studies focus on time trends, prevalence, and predicting factors—all of which are frequently difficult or impossible to change while a patient is waiting in the area [30]. In research conducted by Dziadzko et al. [31], clinically significant changes in anxiety levels were noted in 24.7% of patients during their waiting period. Upon reviewing the literature, we found no studies examining both acute preoperative anxiety in the holding area and its impact on postoperative pain in patients undergoing lumbar disc surgery. This study was designed in response to recent reports indicating that preoperative anxiety rates in spinal surgeries performed under general anesthesia can reach up to 87% [32]. Therefore, the aim is to investigate whether routine clinical practices—such as the duration of the preoperative holding area waiting period—independently affect anxiety and subsequent pain levels during the first 24 h postoperatively. Based on the literature and clinical observations, we hypothesize that the length of time spent in the preoperative holding area is significantly associated with higher levels of preoperative anxiety in patients undergoing lumbar disc surgery and that the higher the level of this anxiety, the greater the postoperative pain experienced within the first 24 h after surgery.

## 2. Materials and methods

### 2.1. Participants and study design

The study included sixty patients scheduled for lumbar disc herniation surgery at the Neurosurgery Clinic at Istinye University Gaziosmanpaşa Medical Park Hospital. To control for potential confounding variables that may influence preoperative anxiety—such as the surgeon’s experience, communication skills, level of preoperative information provided, and the severity of the surgery—only patients undergoing lumbar disc herniation surgery performed by the same surgeon were included in the study. Due to their discontinuation of the offered tests, twelve patients were not included in the study. Forty-eight participants who met the inclusion criteria and provided written and verbal informed consent were included in the study. The study was approved by the Istinye University Human Research Ethics Committee on August 1, 2024 (Protocol No. 24–137, meeting number 2024/06). The Helsinki Declaration on Human Rights has guided the planning of the project. Inclusion criteria were age ≥ 18 years, literacy and ability to communicate, having undergone lumbar disc herniation surgery, and voluntary participation in the study. Exclusion criteria for the study were use of alcohol or drugs, history of psychiatric illness, central nervous system disease, use of psychotropic drugs, pregnancy, visual or hearing impairment, and not providing written and verbal consent.

Patients included in the study were asked to complete the Amsterdam Preoperative Anxiety and Information Scale (APAIS), Visual Analog Scale (VAS), and State-Trait Anxiety Inventory (STAI) scales when they were moved to the preoperative holding area. At the end of the waiting period, patients were asked to complete the APAIS and State Anxiety Inventory-State (STAI-S) scales again. After the surgery, they were asked to fill out the VAS. Additionally, the time spent in the preoperative holding area and the required tramadol dose for the patient’s current pain were recorded.

The power analysis for this study was based on the findings of Dziadzko et al. [31], who reported clinically significant changes in preoperative anxiety levels in 24.7% of patients during their stay in the surgical holding area. Assuming a moderate effect size (Cohen’s d ≈ 0.5), with an alpha level of 0.05 and statistical power (1-β) of 0.80, the minimum required sample size for a paired-sample comparison (e.g., prewaiting vs. postwaiting anxiety levels) was calculated to be approximately 34 participants. Considering the possibility of missing data and the inclusion of multivariate analyses such as linear regression, a total of 48 participants were recruited to ensure adequate statistical power and robustness of the findings.

### 2.2. Outcome measures

#### 2.2.1. Sociodemographic data form

This form, developed by researchers, was used to collect data on patients’ age, occupation, education level, and marital status.

#### 2.2.2. The Amsterdam Preoperative Anxiety and Information Scale (APAIS)

One instrument used to measure preoperative anxiety is the APAIS, which was created by the Moerman group in 1996 [33]. Aykent et al. conducted validity and reliability research in Turkish [34]. A 5-point Likert scale is used to rate the six items on the scale, with 1 denoting never, 2 little, 3 moderate, 4 a lot, and 5 always. The APAIS comprises three subscales: desire for information about the procedure, anesthesia-related anxiety, and surgical-related anxiety. The scale has a minimum possible score of 6 and a maximum possible score of 30 [33,34].

#### 2.2.3. The State-Trait Anxiety Inventory (STAI)

Spielberger et al. [35] created the STAI in 1970. This 40-item Likert-type scale consists of two subscales: 20 items assessing state anxiety and 20 items assessing trait anxiety. Respondents use four alternatives to indicate how strongly they feel and behave: “not at all,” “somewhat,” “very much,” and “completely.” Anxiety levels are correlated with greater scores and lower scores, respectively. Öner et al. established the scale’s reliability and validity in Turkish [35,36].

#### 2.2.4. The Visual Analog Scale (VAS)

Participants’ pain levels were assessed using the VAS, which rates pain intensity from 0 to 10, was used to evaluate participants’ pain levels. The scale features two endpoints: “No pain at all (0)” and “Severe pain (10).” Participants were asked to mark their current pain intensity on this line [37].

#### 2.2.5. Assessment of preoperative holding area time duration

The preoperative waiting time was defined as the time interval between the patient’s arrival at the preoperative holding area and their entry into the operating room. This duration was calculated in minutes based on the time records documented in the nurse observation forms. By relying on objective time records, individualized waiting times were accurately determined for each participant and used in subsequent analyses.

### 2.3. Statistical analysis

IBM SPSS Statistics 29.0.2.0 (IBM Corp., Armonk, NY, USA) were used to analyze the data and the data distribution was evaluated using the Shapiro–Wilk test. For continuous variables with a normal distribution, descriptive statistics were displayed as mean ± standard deviation; for nonnormally distributed variables, they were displayed as median (minimum-maximum). Frequencies and percentages were used to report categorical variables. To assess variations in measurements made at various times within the same group, the Wilcoxon signed-rank test was employed. Spearman’s correlation technique was used to examine correlations between continuous variables. To account for multiple comparisons, Bonferroni correction was applied, and the adjusted threshold for statistical significance was set at p < 0.0014. Additionally, multiple linear regression analyses were conducted, in which age, sex, and education level were included as covariates to control for potential confounding effects.

## 3. Results

The demographic analysis of the 48 participants included in the study revealed that the mean age of the participants was 47.79 ± 2.10. Regarding sex distribution, the number of female participants was 26, while the number of male participants was 22. Among the participants, 25 had a history of previous surgery, and 10 of them had undergone spinal surgery. The average time spent by the participants in the preoperative holding area was calculated as 12.97 ± 0.75 min. Descriptive statistics for other variables are also presented in Table 1.

According to the Mann–Whitney U test results, there was a statistically significant difference between sexes in STAI-S (prewait) scores (Z = −2.054, p = 0.040) and STAI-S (postwait) scores (Z = −2.126, p = .033). No significant sex differences were found for the other variables (p > 0.05).

A Kruskal–Wallis test was used to examine the differences in anxiety, pain, and related measures across educational level groups. The analysis revealed no statistically significant differences among the education groups in terms of APAIS (prewait) (χ^2^(2) = 3.503, p = 0.173), APAIS (postwait) (χ^2^(2) = 5.181, p = 0.075), preoperative VAS (χ^2^(2) = 2.275, p = 0.321), postoperative VAS (χ^2^(2) = 1.860, p = 0.395), tramadol dosage (χ^2^(2) = 2.233, p = 0.327), STAI-State (prewait) (χ^2^(2) = 3.398, p = 0.183), STAI-State (postwait) (χ^2^(2) = 5.941, p = 0.051), or STAI-Trait (χ^2^(2) = 1.855, p = 0.396). Although STAI-State (postwait) approached statistical significance, the difference was not significant at the p < 0.05 level.

Only the APAIS scores showed a significant difference (p = 0.013) when the preoperative and postoperative VAS scores, STAI-S, and APAIS scores were reanalyzed for the time interval between arriving at the preoperative holding area and being transferred to the operating room. Twenty-six subjects showed a substantial increase in their APAIS scores following the waiting time. The ratings on the other scales showed no statistically significant differences (p > 0.05). Table 2 displays the findings of the analysis.

Spearman correlation analysis was conducted to assess the relationships between time spent in the preoperative holding area, anxiety scores, and pain levels. After applying the Bonferroni correction for multiple comparisons (adjusted significance threshold: p < 0.0014), some correlations remained statistically significant. A strong positive correlation was found between the preoperative holding area duration and APAIS (postwait) scores (r = 0.629, p < 0.001), as well as a significant correlation with STAI-S (postwait) scores (r = 0.482, p < 0.001). The results of the correlation analysis are presented in Table 3.

A multiple linear regression analysis was conducted to examine the predictors of the change in APAIS scores (APAIS difference). The model was statistically significant (F (9, 38) = 3.722, p = 0.002), accounting for approximately 34.3% of the variance in APAIS score changes (Adjusted R^2^ = 0.343). The duration spent in the preoperative holding area was found to be a strong and significant predictor of increased APAIS scores (B = 0.516, p < 0.001). Additionally, the preoperative STAI-State (prewait) score was negatively associated with APAIS difference (B = −0.213, p = 0.008). Other variables, including age, sex, prior surgery history, education, preoperative pain levels, and STAI-Trait, did not significantly predict APAIS score changes. The regression data are presented in Table 4.

## 4. Discussion

This study assessed the relationship between preoperative anxiety, postoperative pain, and the duration of stay in the preoperative holding area. It was found that patients who waited longer in the preoperative holding area had higher levels of surgical concern and anxiety. However, no significant relationship was found between preoperative anxiety levels and postoperative pain scores or the amount of analgesic used.

Preoperative anxiety is a common issue that can negatively affect surgical outcomes. Studies have shown that significantly reducing preoperative anxiety levels surgical recovery [38]. In a variety of surgical settings, preoperative anxiety has been demonstrated to have a substantial impact on postoperative pain outcomes. Higher preoperative anxiety levels have been linked to more severe postoperative pain and greater demands for analgesics, according to several research studies [39,40]. Patients undergoing heart surgery who experienced moderate to severe preoperative anxiety exhibited a larger demand for morphine and higher pain levels than those who experienced mild anxiety [39]. In a randomized controlled trial, Yadav et al. [41] demonstrated that preoperative administration of 300 mg of pregabalin effectively reduced preoperative anxiety and postoperative pain, as well as postoperative opioid consumption. Similarly, Tadesse et al. [9] highlighted that reducing anxiety can significantly influence postoperative pain and is associated with substantial postoperative analgesic use. Adogwa et al. [42] further demonstrated a similar relationship in their study involving patients undergoing cervical spinal surgery.

The literature has shown that prolonged preoperative waiting time is associated with increased anxiety levels. For example, a 2024 study by Wu et al. discovered that patients’ feelings of discomfort and anxiety rose with preoperative waiting time [11]. Similarly, 57.9% of patients who remained in the holding area for 30 min or more reported experiencing significant anxiety, according to a 2020 study by Prayogi et al. [43]. Consistent with these findings, our study also determined that prolonged preoperative waiting time is associated with increased anxiety levels. Waiting times can be shortened by optimizing surgical schedules and minimizing unnecessary delays. This approach may help prevent anxiety and the severity of associated postoperative complications. Consequently, more effective surgical interventions can be achieved, contributing positively to patients’ quality of life.

Although the average preoperative holding time was relatively short—approximately 12.97 ± 0.75 min—a statistically significant increase was observed in APAIS scores during this period. This finding suggests that even brief delays before surgery can elicit a measurable increase in patient anxiety. Clinically, this is notable because APAIS is a sensitive tool specifically designed to detect preoperative anxiety and informational needs in surgical settings. According to Moerman et al. [33], a change of 4 or more points in the APAIS score is considered clinically meaningful, indicating a shift from mild to moderate anxiety levels. In our sample, the observed increase in APAIS scores approached or exceeded this threshold in over half of the participants, which underscores the psychological impact of waiting even for a short duration. Other studies have similarly found that perceived waiting time and uncertainty in the preoperative period contribute significantly to elevated anxiety, potentially affecting perioperative outcomes such as pain perception and patient cooperation (2, 44). Therefore, our findings highlight the importance of not only minimizing waiting durations but also improving the quality of the waiting experience through patient education and environmental adjustments.

In this study, although a statistically significant increase was observed in APAIS scores at the end of the preoperative waiting period, no significant change was found in STAI-S scores. This discrepancy may stem from the differences in the measurement scope and sensitivity of the scales used. The APAIS is a brief, surgery-specific instrument designed to assess preoperative anxiety and the need for information, with greater sensitivity to the immediate and context-specific aspects of anxiety in the surgical setting [33]. In contrast, the STAI-S measures general state anxiety and reflects broader aspects of anxiety that are less dependent on situational factors [35]. Comparative studies evaluating preoperative anxiety using both APAIS and STAI-S have reported that STAI-S, being designed for identifying clinically significant anxiety states, may require higher thresholds for detecting meaningful changes. Therefore, in our study, while the waiting period may have directly increased anxiety related to the surgery—captured by the APAIS—this change may not have been substantial enough to significantly affect the more general anxiety levels measured by the STAI-S.

In our study investigating the relationship between preoperative waiting time, anxiety levels, postoperative pain, and the required analgesic dosage to alleviate this pain, we found—consistent with the existing literature—that prolonged preoperative waiting time is associated with increased preoperative anxiety. However, no significant relationship was found between preoperative anxiety and postoperative pain in our study. This may be attributed to the relatively small sample size. In line with these findings, it is crucial to implement strategies aimed at reducing preoperative waiting times, anxiety, and the associated negative outcomes. Enhancing waiting areas with calming elements such as music, warm blankets, or aromatherapy may improve patient comfort [19–24]. Providing patients with clear and consistent information about the surgical process can also reduce uncertainty and anxiety [17]. Future studies should examine how well non-pharmacological therapies, including mindfulness practices or breathing and relaxation exercises, can lower preoperative anxiety. Additionally, larger-scale studies examining the impact of sociodemographic factors and previous surgical experiences on anxiety levels could provide deeper insights. Integrating these approaches into clinical practice could not only increase patient satisfaction but also improve postoperative outcomes by reducing pain and analgesic needs while strengthening the patient-physician relationship.

There are various limitations to our investigation. First, there are only a few patients in this single-center trial. As a result, it may be subject to selection bias, which can limit the generalizability of our findings. Patients with diagnosed psychiatric disorders were excluded from the study; however, individuals with undiagnosed anxiety or depression might have been overlooked due to the use of self-report scales. This could affect the results and create a false correlation in the statistical analysis. Furthermore, the generalizability of our findings is limited due to the homogeneous nature of the study sample. All participants underwent lumbar spine surgery and received tramadol for postoperative pain management, which restricts the applicability of our results to broader surgical populations or to those managed with alternative analgesics. Previous studies have shown that anxiety and pain perception can vary significantly depending on the type of surgery (e.g., cardiac vs. orthopedic) and the invasiveness of the procedure [45]. Moreover, different analgesics may interact with psychological states in distinct ways, potentially influencing both perceived pain and recovery experience [46]. Thus, while our findings provide valuable insight into spinal surgery patients, further research involving diverse surgical populations and varying pain management protocols is necessary to confirm and extend the applicability of our conclusions. Our results are in line with previous research; however, we believe these findings could be strengthened by additional metaanalyses and multicenter randomizes controlled trials with larger sample sizes.

## 5. Conclusion

Our findings show that anxiety levels are positively correlated with preoperative waiting times. These findings emphasize the importance of treating preoperative anxiety to improve surgical outcomes and patient satisfaction. Reducing waiting times, implementing preoperative anxiety management strategies, and providing patients with information may also help decrease postoperative complications. Further multicenter research is needed to confirm these results and explore focused strategies to improve perioperative care.

## Figures and Tables

**Table 1:** Sociodemographic characteristics and preoperative/postoperative anxiety and pain measurements of participants.

	n=48

Age	47.79±2.10

Sex	
Female	26
Male	22

History of previous surgery	
Surgical group	25
Nonsurgical group	23

History of previous spinal surgery	
Surgical group	10
Nonsurgical group	38

Education level	
Primary school	20
High school	14
University	14

Preoperative holding area Duration (minutes)	12.97±0.75

APAIS (prewait)	13.00 (6.00–26.00)

APAIS (postwait)	15.50 (6.00–28.00)

STAI-S (prewait)	32.00 (21.00–66.00)

STAI-S (postwait)	32.00 (20.00–72.00)

STAI-T	25.00 (20.00–60.00)

Preoperative VAS	6.00 (5.00–9.00)

Postoperative VAS	6.00 (5.00–9.00)

Tramadol for postoperative pain relief	200 mg (100–300 mg)

APAIS (The Amsterdam Preoperative Anxiety and Information Scale), STAI (State-Trait Anxiety Inventory), VAS (Visual Analog Scale). For parametric data, mean ± SD (mean ± standard deviation), for nonparametric data, median (min–max) values, and for categorical data, frequency values are presented.

**Table 2:** Wilcoxon signed-rank test results for changes in APAIS and STAI scores from preoperative to postwaiting room, and pre- and postoperative changes in VAS score.

	Negative ranks	Positive ranks	Ties	p
APAIS (postwait)-APAIS (prewait)	10^a^	26^b^	12^c^	0.013*
Postop VAS-Preop VAS	18^d^	20^e^	10^f^	0.621
STAI-S (postwait)-STAI-S (prewait)	21^g^	20^h^	7^i^	0.305

Wilcoxon signed-rank test.

* p < 0.05. APAIS (The Amsterdam Preoperative Anxiety and Information Scale), STAI-S/T (State-Trait Anxiety Inventory State/Trait), VAS (Visual Analog Scale).

a APAIS (postwait)< APAIS (pre-wait),

b APAIS (postwait)> APAIS (prewait),

c APAIS (postwait)= APAIS (prewait),

d Postop VAS< Preop VAS,

e Postop VAS> Preop VAS,

f Postop VAS=Preop VAS,

g STAI-S (postwait)< STAI-S (prewait),

h STAI-S (postwait)> STAI-S (prewait),

i STAI-S (postwait)=STAI-S (prewait).

**Table 3:** Correlation coefficients and p-values for waiting room duration, APAIS, STAI, VAS scores, and postoperative tramadol usage.

		Preoperative holding area duration (minutes)	APAIS (prewait)	APAIS (postwait)	Preop VAS	Postop VAS	STAI-S (prewait)	STAI-S (postwait)	STAI-T	Tramadol for postoperative pain relief
Preoperative holding area duration (minutes)	Coef.	1.000	0.288*	0.626**	0.209	0.309*	0.196	0.482**	0.405**	0.282
APAIS (prewait)	Coef.	0.288*	1.000	0.669**	0.431**	0.192	0.760**	0.585**	0.539**	0.124
APAIS (postwait)	Coef.	0.626**	0.669**	1.000	0.470**	0.237	0.552**	0.757**	0.575**	0.208
Preop. VAS	Coef.	0.209	0.431**	0.470**	1.000	0.123	0.296*	0.280	0.149	0.226
Postop. VAS	Coef.	0.309*	0.192	0.237	0.123	1.000	0.112	0.163	0.102	0.153
STAI-S (prewait)	Coef.	0.196	0.760**	0.552**	0.296*	0.112	1.000	0.761**	0.790**	0.187
STAI-S (postwait)	Coef.	0.482**	0.585**	0.757**	0.280	0.163	0.761**	1.000	0.792**	0.286*
STAI-T	Coef.	0.405**	0.539**	0.575**	0.149	0.102	0.790**	0.792**	1.000	0.306*
Tramadol for postoperative pain relief	Coef.	0.282	0.124	0.208	0.226	0.153	0.187	0.286*	0.306*	1.000

Spearman Correlation Test. Coef.: Coefficient., APAIS (The Amsterdam Preoperative Anxiety and Information Scale), STAI-S/T (State-Trait Anxiety Inventory State/Trait), VAS (Visual Analog Scale), Preop.: (Preoperative), Postop.: (Postoperative).

* p < 0.05,

** p < 0.0014 (Bonferroni-corrected significance threshold)

**Table 4:** Regression analysis predicting change in APAIS scores.

Predictor variables	B	SE B	Beta	t	p
Constant	−15.970	5.367		−2.975	0.005*
Age	0.031	0.046	0.089	0.675	0.504
Sex	1.594	1.340	0.157	1.190	0.242
Previous Surgery	2.111	1.260	0.208	1.676	0.102
Previous spinal surgery	1.400	1.577	0.112	0.888	0.380
Education	0.800	0.771	0.131	1.038	0.306
Preoperative holding area duration (minutes)	0.516	0.132	0.525	3.902	<0.001**
Preop. VAS	0.606	0.583	0.153	1.039	0.305
STAI-Trait	0.145	0.107	0.247	1.356	0.183
STAI-State (prewait)	−0.213	0.076	−0.528	−2.796	0.008*

Multiple Linear Regression. B = Unstandardized Coefficient; SE B = Standard Error of B; Beta = Standardized Coefficient; t = t-value; p = p-value. APAIS (The Amsterdam Preoperative Anxiety and Information Scale), STAI-S/T (State-Trait Anxiety Inventory State/Trait), VAS (Visual Analog Scale), Preop.: (Preoperative).

* p < 0.05,

** p < 0.001
